# A Rare Genetic Intersection: Down Syndrome With Coexisting Spinal Muscular Atrophy

**DOI:** 10.7759/cureus.67243

**Published:** 2024-08-19

**Authors:** Bhavna Gupta, Madiha Mohamed, Aman Sohal, Theekshitha K Kamalakannan, Batool Balouch, Funda Cipe

**Affiliations:** 1 Department of Pediatrics/Specialist Pediatrics, Al Qassimi Women’s and Children’s Hospital, Sharjah, ARE; 2 Department of Pediatrics, Al Qassimi Women's and Children's Hospital, Sharjah, ARE; 3 Departments of Pediatrics, Al Qassimi Women and Children’s Hospital, Sharjah, ARE; 4 Department of Pediatric Neurology, Great Ormond Street Children’s Hospital, London, GBR; 5 Department of Pediatrics, Al Qassimi Women’s and Children’s Hospital, Sharjah, ARE; 6 Department of Pediatric, Al Qassimi Women's and Children's Hospital, Sharjah, ARE

**Keywords:** pediatrics, pediatric neurology, genetic disorder, spinal muscular atrophy, down syndrome

## Abstract

Down syndrome (DS), characterized by trisomy of chromosome 21, and spinal muscular atrophy (SMA), an autosomal recessive neuromuscular disorder, are individually recognized distinct entities. Their co-occurrence in clinical practice is rare and has not been extensively reported.

We present a case of a three-month-old, female child who presented with respiratory failure necessitating intubation. Due to typical facial features and congenital heart disease, DS was confirmed with chromosomal analysis. However, subsequent recurrent chest and bloodstream infections, failure to extubate, and laboratory abnormalities raised the suspicion of accompanying immune disorder with DS. To investigate this, whole exome sequencing analysis was sent, and it revealed a homozygous pathogenic mutation in the SMA type 1 gene in the patient.

This rare intersection of two unique genetic conditions presents diagnostic challenges due to overlapping clinical features like hypotonia and delay in motor skills, which can be progressive in both situations. Additionally, the clinical trajectory, therapeutic interventions, and outcomes are variable for both conditions and a lack of guidelines for the management of two concurrent genetic conditions, such as in our patient, can pose a challenge for clinicians.

Hence, this case report underscores the importance of comprehensive clinical and diagnostic evaluation in individuals with syndromic features and the need for heightened vigilance for concurrent rare genetic conditions that add to the complexity of the disease and may impact clinical outcomes, management, and counseling for the family.

## Introduction

Down syndrome (DS), medically referred to as trisomy 21, is commonly identified at birth due to its distinctive facial features and associated anomalies [1]. A reduction in brain growth and developmental delays marks this genetic condition. Individuals affected often exhibit decreased muscle tone, impaired motor coordination, and a slower progression in both motor skills and cognitive abilities [2]. Challenges may also hinder language acquisition in auditory memory. Moreover, individuals with DS are prone to various neurological conditions, including epilepsy, autism, and attention-deficit/hyperactivity disorder (ADHD) [1]. It may also cause complications like strokes (potentially linked to congenital heart defects), cervical spinal cord compression (associated with anomalies in the atlantoaxial joint), and damage to the basal ganglia [1].

On the other hand, spinal muscular atrophy (SMA) is a rare neuromuscular disorder resulting from the deletion or mutation of the survival motor neuron 1 (SMN1) gene. This condition is characterized by the gradual degeneration of motor neurons in the anterior horn of the spinal cord, leading to muscle atrophy, weakness, and eventual paralysis. Unlike DS, SMA typically does not present with cognitive deficits [3].

The co-existence of SMA with DS is extremely rare and here we highlight a case who needed prolonged ventilation at three months of age with recurrent infections. With suspicion of an underlying etiology of associated immunodeficiency, beyond the diagnosis of DS, Whole Exome Sequencing was done, which was suggestive of homozygous pathogenic deletion of exons 7 and 8 of the SMN1 gene, confirmed by MLPA analysis.

Advances in molecular genetics and cytogenetics have enabled the diagnostic confirmation of the spectrum of complex or atypical clinical presentations of genetic disorders [4]. The co-occurrence of two independently inherited genetic diseases represents a unique pathological scenario, exacerbated by the cumulative effects of different genetic disorders [5]. This significantly increases clinical complexity, leading to poorer health outcomes, higher healthcare costs, and more intricate clinical management [6].

In this case study, genetic analysis identifies dual genetic associations in a single patient, one caused by chromosomal aneuploidy and the other by gene deletion [7]. The suspicion of being affected by a coexistent rare genetic condition arises when signs and symptoms do not align with the primary diagnosis. This has significant implications for appropriate genetic counseling, determining accurate prognoses, and organizing optimal long-term follow-up care [4].

## Case presentation

A three-month-old infant, presented to our hospital, with respiratory distress and hypoxia and needed admission to the Pediatric ICU (PICU). She was born to consanguineous parents at 35 weeks of gestation in another hospital through normal vaginal delivery, with a low birth weight for gestational age of 2.08 kg. The neonatal period was uneventful except for hypoglycemia on the first day and moderate Patent Ductus Arteriosus (PDA), diagnosed using echocardiography. However, there were no records of any genetic studies done at birth for the baby. The mother also reported feeding issues since birth. There was no family history of any genetic disorder.

In the PICU on initial examination, she had dysmorphic features (depressed nasal bridge, upslanting eyes, and low-set ears ) consistent with DS, hypotonia, failure to thrive, and respiratory distress. She was initially managed as a case of aspiration pneumonia, with noninvasive ventilation and antibiotics. Later due to clinical deterioration, the baby was intubated and ventilated, for more than one month, with several failed attempts at extubation. Hence, further workup was initiated, including viral panels, cultures, creatinine phosphate kinase, and investigations for immunodeficiency. Transcatheter PDA closure was also done to support respiration

Since DS is known to be associated with immunodeficiency and our patient exhibited many suggestive clinical and laboratory findings such as failure to thrive, recurrent lung infection, oral candidiasis, persistent lymphopenia, pseudomonas infection in endotracheal tube culture, CMV infection (CMV PCR DNA positive), and low lymphocyte subsets counts, combined immunodeficiency was highly suspected. She was started on antivirals, antifungal, and antibacterial (sulfamethoxazole-trimethoprim) prophylaxis. Subsequently, the child's condition improved, and she was discharged after staying in the PICU for more than a month, awaiting genetic results.

**Figure 1:**
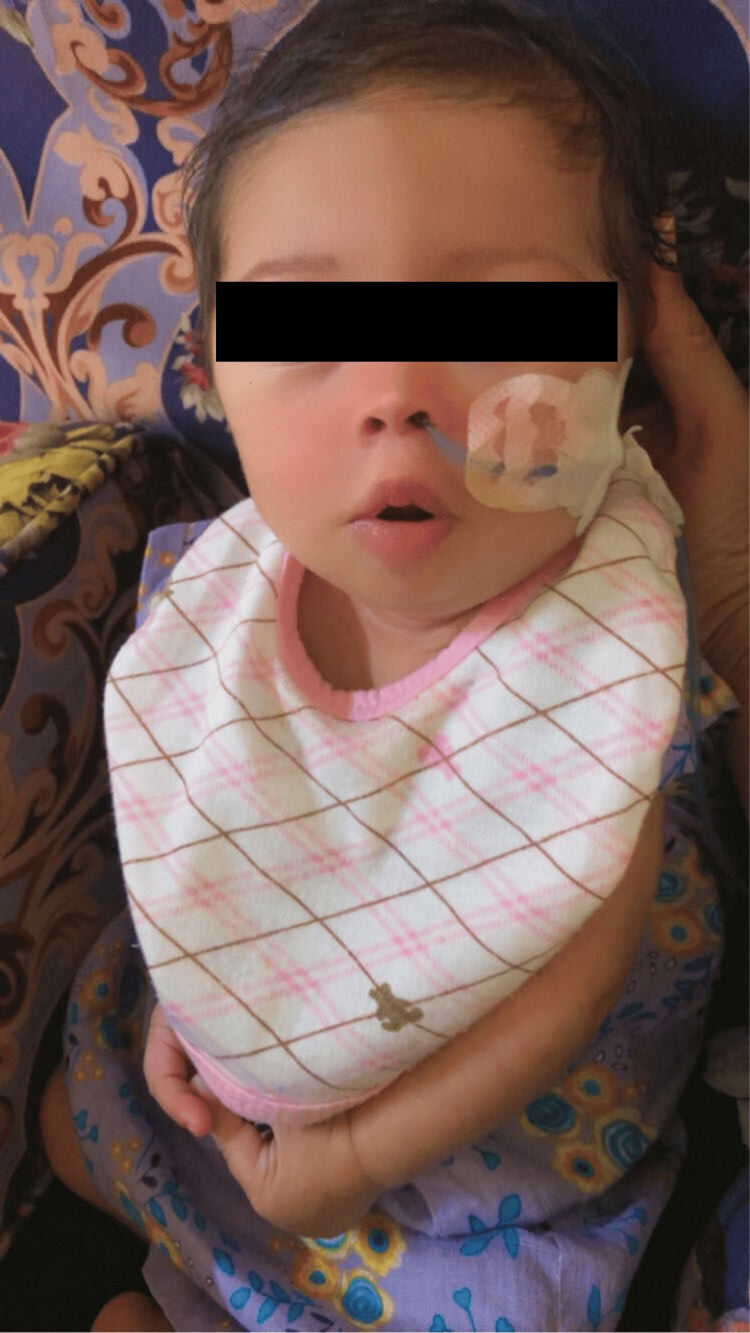
The infant with Down syndrome. A nasogastric tube was inserted because of feeding difficulties.

Genetic studies, including initially karyotype for confirming DS were sent. Later, because basic immunological tests did not diagnose primary immunodeficiency accurately, whole exome sequencing (WES) was sent to rule out accompanying genetic mutation causing combined immunodeficiency. Trisomy 21 was confirmed in both karyotypes and WES (Figure 1).

Surprisingly, WES also revealed a possible concurrent homozygous deletion of exons 7 and 8 of the SMN1 gene, which was confirmed by multiplex ligation-dependent probe amplification (MLPA) analysis.

Hence, a diagnosis of DS with co-existing SMA type 1 was established. Multidisciplinary teams, including pediatric neurologists, pediatric pulmonologists, geneticists, and rehabilitation specialists, were involved in the management.

## Discussion

DS is one of the most common chromosomal disorders and a common cause associated with intellectual disability. It is primarily caused by trisomy of chromosome 21, with a wide range of phenotypic variations, and multiple systemic complications as part of the syndrome [8].

It’s well established that the clinical profile of recurrent infections, autoinflammatory diseases, and hematologic malignancies in DS can correspond to a possible phenotype of combined immunodeficiency [9]. It has been studied that T-cell development and function are variable in cases of DS. T-cell receptor excision circles (TREC) count and recent thymic emigrants that can be used to characterize thymic output are decreased in children with DS [10]. In our patient, CD4 and CD19 cells were found to be very low however, we were unable to perform lymphocyte functions and TRECs due to laboratory logistics. Since there are rare case reports together with DS and severe combined immunodeficiency, we wanted to rule out primary immunodeficiencies by WES [11].

However, WES identified a concurrent homozygous deletion of exons 7 and 8 of the SMN1 gene, revealing the diagnosis of SMA, confirmed on MLPA analysis.

SMA encompasses a group of genetic disorders that can damage and destroy motor neurons, the specialized nerve cells in the brain and spinal cord. The most common form of SMA is attributed to a mutation or absence of the SMN1 gene located on chromosome 5q. This gene is typically responsible for producing a protein essential for motor neuron function [12]. The loss of motor neurons in the spinal cord leads to weakness and wasting of the skeletal muscles, which is further complicated by difficulties with walking, swallowing, and breathing [12].

From a genetic standpoint, our patient presented with two distinctly inherited conditions. DS is characterized by a chromosomal abnormality involving an additional chromosome (trisomy 21) [13]. Alternatively, SMA is inherited in an autosomal recessive manner and results from mutations in the SMN1 gene [3]. As a consequence, the risk of recurrence differs for each condition. DS typically occurs as a random event during the formation of reproductive cells in a parent, thus carrying a low risk of recurrence [14]. In contrast, SMA poses a 25% risk in each subsequent pregnancy due to its autosomal recessive inheritance pattern. Hence this genetic symbiosis can implicate prognostication and counseling and emphasizes the importance of initiating a multidisciplinary approach in the management and follow-up of these cases. 

Moreover, the occurrence of both SMA and DS together is exceedingly rare, as evidenced by our review of the literature which yielded only two reported cases. In one instance, SMA was identified during the neonatal period, while in another case, SMA was diagnosed in a DS child at the age of six [13,14].

In terms of overlapping clinical features in DS and SMA, in cases of DS, it is theorized that hypotonia and lax ligaments may contribute to motor delays [15]. Conversely, SMA involves the progressive loss of nerve cells in the spinal cord known as lower motor neurons or anterior horn cells, leading to muscle weakness, wasting, and hypotonia.

Therefore, in the current era of medicine, advancements in genomics, particularly in the identification of rare diseases, have necessitated a shift in physicians' perspectives on various conditions. This shift impacts not only clinical evaluation but also the range of diagnostic investigations offered to patients. Clinicians must be increasingly vigilant in their assessment of rare diseases particularly in consanguineous populations, as the outcomes of these evaluations influence management plans, including the involvement of multidisciplinary teams, counseling, and follow-up care. Hence, this case report underscores the importance of conducting thorough investigations when a patient's clinical presentation cannot be fully accounted for by the underlying condition.

Additionally, recent years have seen significant improvements in the standard care and pharmacological treatments for both SMA and DS. The development of treatment modalities, such as gene therapy for SMA, has notably enhanced the quality of life for affected individuals and their families. Therefore, physicians must stay alert to these conditions, as timely intervention can lead to better outcomes and improved patient well-being.

## Conclusions

This case report highlights the uncommon co-occurrence of two distinct genetic conditions in a single patient. It underscores the importance of maintaining a high index of suspicion when a clinical phenotype does not align entirely with the initial genetic diagnosis. Moreover, the coexistence of DS and SMA poses unique challenges due to overlapping clinical features and compounded genetic risks. While the literature suggests a low risk of recurrence, early diagnosis is crucial, as this could not only have implications on therapeutic outcomes, but also on the anticipation of potential complications, the involvement of multidisciplinary teams, and the provision of genetic counseling to the family. Further research is warranted to explore the genetic and clinical interactions for such conditions, for potential future therapeutic approaches and improving patient outcomes!
